# Respiratory Syncytial Virus-associated hospitalization in premature infants who did not receive palivizumab prophylaxis in Italy: a retrospective analysis from the Osservatorio Study

**DOI:** 10.1186/s13052-016-0252-9

**Published:** 2016-04-26

**Authors:** Michela Silvestri, Francesca Marando, Anna Maria Costanzo, Umberto di Luzio Paparatti, Giovanni A. Rossi

**Affiliations:** Pediatric Pulmonology and Allergy Unit and Cystic Fibrosis Center, Istituto Giannina Gaslini, Genoa, Italy; AbbVie, Medical Department, Campoverde di Aprilia (LT), Campoverde di Aprilia (Latina), Italy

**Keywords:** Acute lower respiratory tract infection, Bronchiolitis, Gestational age

## Abstract

**Background:**

Due to different social and epidemiological factors, the eligibility criteria to receive palivizumab prophylaxis may be different between countries, especially in “otherwise healthy” late preterm infants.

**Methods:**

We analyzed an Italian database of young children referred to emergency departments for acute lower respiratory tract infection (ALRI) during the RSV season over a four year period, when the use of palivizumab as prophylaxis for RSV disease was not widespread in premature infants. The demographic and environmental characteristics and the RSV positivity (RSV^+^) in hospitalized and not-hospitalized patients were compared. In the data analysis we divided children according to their chronologic age (age) and their week gestational age (wGA).

**Results:**

Out of the 100 children evaluated, 68 were infants (≤12 month-age): 7.5 and 20.6 % were in the <29 and 29- < 32 wGA groups respectively, and 72.0 % in the 32- < 35 wGA group. Positive hospitalized-to-not-hospitalized ratios were found in all three wGA groups, progressively decreasing (from 4.0 to 1.2), with increasing wGA (*p* = 0.35). The percentage of hospitalized infants that were also RSV^+^ was also progressively decreasing (from 40.0 to 28.6 % and 18.4 %) with increasing wGA (*p* = 0.43). In the >12 month-age group (*N* = 32), there was positive hospitalized-to-not-hospitalized ratio only in the <29 wGA group with a low RSV^+^ frequency (<29 %) in all wGA groups. In the ≤12 month-age group, 41 infants were evaluated with a ≤6 month-age and 27 with a >6–12 month-age. A positive hospitalized-to-not-hospitalized ratios was found in all wGA groups in ≤6 month-age infants, despite a low RSV^+^ frequency in the 29- < 32 and 32- < 35 wGA group. In the >6-12 month-age group, all infants with a <29 and 29- < 32 wGA were hospitalized with a relatively high RSV^+^ frequency whilst the 32- < 35 wGA group showed a negative hospitalized-to-not-hospitalized ratio with a lower RSV^+^ frequency.

**Conclusions:**

The hospitalized-to-not-hospitalized ratios and RSV^+^ frequency in the first 12 months of age in infants born prematurely confirm the vulnerability of these children for clinically important RSV infection, most notably in the <32 wGA category.

## Background

Respiratory syncytial virus (RSV) is the single most important cause of acute lower respiratory tract infection (ALRI) in infants and young children worldwide and, in these target populations, bronchiolitis due to RSV is associated with significant morbidity and, sometimes, mortality [1]. In industrialized countries there is a common belief that RSV is primarily a serious agent in infants and the disease is generally less common and severe in older children [1–3]. Almost all children are infected by RSV by 2 years of age, 60–70 % are infected in the first year of age and, of these, 2–3 % are hospitalized for bronchiolitis induced by RSV [3–5]. Some children are at risk for disease progression to respiratory failure, mechanical ventilation, and intensive care unit management. In a study performed in Switzerland over a 4-year period (2001–2005) it was shown that RSV-induced bronchiolitis led to the intermediate or intensive care admission of approximately 1–2 % of each annual birth cohort [6]. In a revision of data collected from 24 pediatric intensive care units (PICUs) in France, it was found that 76 % of the 467 young children hospitalized for bronchiolitis were RSV positive: over one-third required noninvasive ventilation and/or mechanical ventilation, and six infants died [7]. To date, there is no effective treatment for RSV-induced bronchiolitis: the mainstay of therapy is supportive care and the possible role of any pharmacological approach is still debated. Environmental preventive measures minimize viral transmission in hospital, in the outpatient setting and at home, whilst the only pharmacological prophylaxis is based on passive immunoprophylaxis with palivizumab, the specific anti-RSV humanized monoclonal antibody proven to be effective in reducing the overall hospitalization rate in specific categories of children at risk during the RSV epidemic period [8]. In infants without pre-existing specific medical conditions, the two major factors increasing the risk of severe RSV-induced bronchiolitis are preterm birth and young chronological age at the start of the RSV season [9–11]. Incidence and severity of RSV disease and significance of risk factors can vary substantially from year to year in any one setting [11]. In an effort to ensure optimal balance of benefit and cost, clinical guidelines are country specific and vary with regard to their recommendations for use of palivizumab prophylaxis, most clinical guidelines recommending that premature infants receive prophylaxis at the start of the RSV season. However, because of different social and epidemiological factors, the eligibility criteria to receive palivizumab prophylaxis at the start of the RSV season are often different between countries, especially when it comes to the “otherwise healthy” late preterm infants [12–15].

Using the historical database of an Italian epidemiological report on hospitalizations during four RSV epidemic seasons [16], we aimed to evaluate the demographic and environmental characteristics and the RSV positivity (RSV^+^) of hospitalized and not-hospitalized children during a period (2000–2004) when palivizumab prophylaxis was not widespread in premature infants. In the data analysis children were divided according to their chronologic age (age) and their week gestational age (wGA).

## Methods

### Populations

**Fig. 1:**
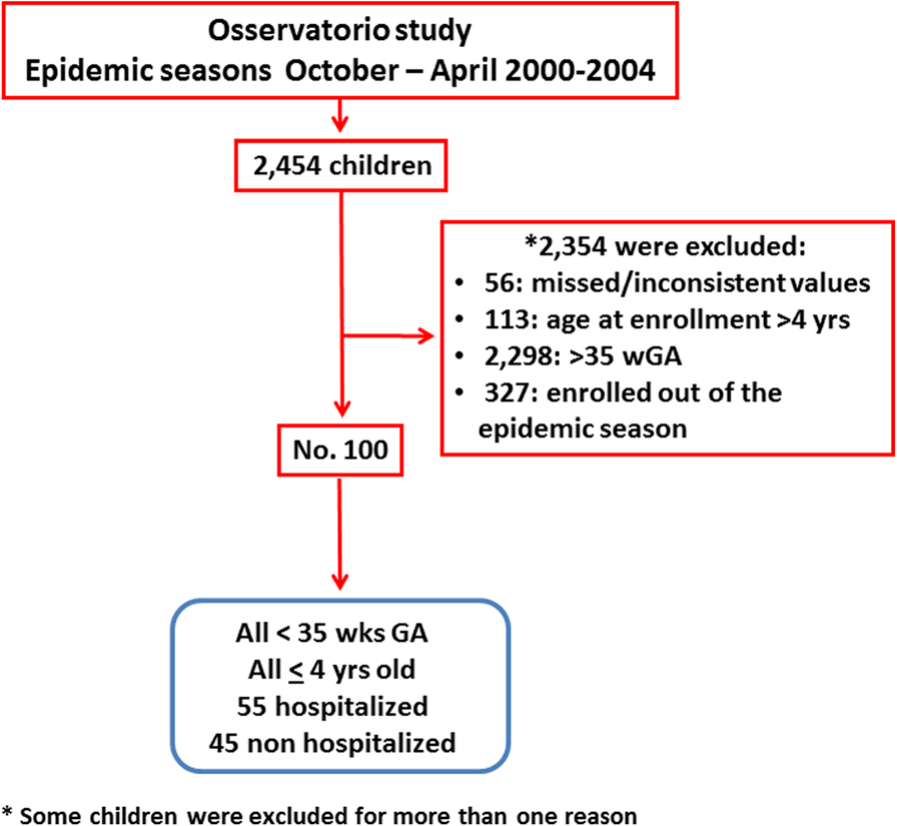
Number of children from the “Osservatorio” database that were firstly analyzed and then included in the sets of analysis, to evaluate the characteristics and the distribution of RSV positivity (RSV+) in hospitalized and not hospitalized children belonging to different wGA groups

The historical database of patients included in the “Osservatorio” study on the epidemiology of RSV infection in children in Italy was analyzed. The study was performed in years when the use of palivizumab in premature infants was not widespread [14]. The inclusion criteria used to select and evaluate patients are reported in Fig. 1. Palivizumab prophylaxis was an exclusion criterion in the study protocol. The study protocol of the Osservatorio study was originally approved by the Institutional Review Board of the Department of Pathology and Laboratory Medicine, University of Parma, Parma, Italy and written informed consent was sought from parents or guardians of all evaluated children. Nasal secretion samples were sent to the microbiology laboratory of each hospital to check for RSV by an immunoenzymatic test (TestPack RSV, Abbott, Italy) within 24h. Other respiratory viruses were not tested.

### The “Osservatorio” database

This study aimed to describe the time-related pattern of RSV epidemics in Italy, across four consecutive epidemics, from 2000 to 2004 [16]. Data were collected on 2241 consecutive children, aged ≤4 years, referred to the emergency departments of 16 tertiary care hospitals (8 centers, during the first season, 14, during the second season and 13, during the third and fourth seasons) for acute respiratory infection, one day of the week (Tuesday), during the expected RSV-epidemiological seasons (October - April). The decision to admit a child to inpatient care was made by local physicians responsible for each child’s care who were not involved in the study. This database collected information on both hospitalized and non-hospitalized children.

### Data analysis

The demographic and environmental characteristics and the distribution of RSV positivity were compared in children born in three different wGA: <29 wGA, 29- < 32 wGA, and 32- < 35 wGA. Children with missing values (i.e. date of birth, wGA at birth, date of hospitalization) were excluded from the analysis (Fig. 1). Age at enrollment, birth order, educational levels of the mother and of the father, day-care attendance, exposure to environmental tobacco smoke, and data pertaining to breast feeding were categorized before the bivariate analysis [11, 16]. Since some information was missing in the original database, the available data for each variable are reported in tables.

### Statistical analysis

Descriptive statistics of the characteristics of the patients were performed and reported in terms of absolute frequencies and percentages for the qualitative variables. Comparison of frequency data was performed by chi-square test or by Fisher’s exact test in case of expected frequencies less than five. All tests were two-sided and P-values less than 0.05 were considered statistically significant. The statistical packages used were the “Statistica release 6” (Stat Soft Corp., Tulsa, OK, USA).

## Results

### Demographic and environmental characteristics of the study population

One hundred children fulfilled the criteria to be analyzed (Fig. 1). The demographic and characteristics of the hospitalized and not hospitalized children, as well as the factors known to increase the risk of hospitalization for RSV-induced ALRI, are shown in Table 1. The risk factors associated with hospitalization for RSV-induced ALRI are reported in Tables 2 and 3, for hospitalized and not hospitalized children, respectively. The presence of other risk factors known to increase the risk of hospitalization for RSV-induced ALRI was not different among the three wGA groups.

**Table 1:** Demographic characteristics and presence of major risk factors for hospitalization for RSV infection in hospitalized and non hospitalized children

	Hospitalized children	Non hospitalized children	p
	[No. (row %)]	[No. (row %)]	
No	55	45	-
Male gender	24 (51.06 %)	23 (48.94 %)	0.51
wGA at birth			
GA < 29 w	7 (77.78 %)	2 (22.22 %)	0.32
GA 29 - <32 w	12 (57.14 %)	9 (42.86 %)	
GA 32 - <35 w	36 (51.43 %)	34 (48.57 %)	
Birth order (No. = 96)			
1	21 (53.85 %)	18 (46.15 %)	0.82
> 1	32 (56.14 %)	25 (43.86 %)	
Educational level of the mother (No. = 32)			
Junior high school	8 (80 %)	2 (20.00 %)	0.25
High school	13 (59.09 %)	9 (40.91 %)	
Educational level of the father (No. = 92)			
Junior high school	22 (46.81 %)	25 (53.19 %)	0.055
High school	30 (66.67 %)	15 (33.33 %)	
CHD	2 (100 %)	0	0.30
BPD	5 (55.56 %)	4 (44.44 %)	0.93
Day-care attendance (No. = 99)			
Yes	4 (36.36 %)	7 (63.64 %)	0.17
No	51 (57.95 %)	37 (42.05 %)	
Exposure to ETS (No. = 98)			
Yes	33 (57.89 %)	24 (42.11 %)	0.68
No	22 (53.66 %)	19 (46.34 %)	
Breast feeding (No. = 24)			
Yes	3 (30 %)	7 (70 %)	0.52
No	6 (42.86 %)	8 (57.14 %)	

*GA* gestational age, *w* weeks, *mo* months, *ETS* environmental tobacco smoke

**Table 2:** Distribution of demographic characteristics and risk factors in hospitalized pts

	<29w GA	29 - <32w GA	32 - <35w GA	p
	[No. (row %)]	[No. (row %)]	[No. (row %)]	
No.	7 (12.73 %)	12 (21.82 %)	36 (65.46 %)	-
Male gender	4 (16.67 %)	5 (20.83 %)	15 (62.50 %)	0.51
Birth order (No. = 53)				
1	4 (19.05 %)	6 (28.57 %)	11 (52.38 %)	0.13
> 1	2 (6.25 %)	5 (15.63 %)	25 (78.13 %)	
Educational level of the mother (No. = 21)				
Junior high school	1 (12.50 %)	2 (25.00 %)	5 (62.50 %)	0.78
High school	1 (7.69 %)	2 (15.38 %)	10 (76.92)	
Educational level of the father (No. = 52)				
Junior high school	4 (18.18 %)	3 (13.64 %)	15 (68.18 %)	0.43
High school	3 (10.00 %)	8 (26.67 %)	19 (63.33 %)	
CHD	0	0	2 (100.00 %)	0.62
BPD	3 (60.00 %)	1 (20.00 %)	1 (20.00)	0.004
Day-care attendance				
Yes	0	0	4 (100.00 %)	0.32
No	7 (13.73 %)	12 (23.53 %)	32 (62.75 %)	
Exposure to ETS				
Yes	4 (12.12 %)	7 (21.21 %)	22 (66.67 %)	0.97
No	3 (13.64 %)	5 (22.73 %)	14 (63.64 %)	
Breast feeding (No. = 9)				
Yes	0	2 (66.67 %)	1 (33.33 %)	0.08
No	0	0	6 (100 %)	

*CHD* congenital heart disease, *BPD* bronchopulmonary dysplasia, *ETS* environmental tobacco smoke

**Table 3:** Distribution of demographic characteristics and risk factors in not hospitalized pts

	<29w GA	29 - <32w GA	32 - <35w GA	p
	[No. (row %)]	[No. (row %)]	[No. (row %)]	
No.	2 (4.44 %)	9 (20.00 %)	34 (75.56 %)	-
Male gender	1 (4.35 %)	4 (17.39 %)	18 (78.26 %)	0.90
Birth order (No. = 9)				
1	0	5 (27.78 %)	13 (72.22 %)	0.34
> 1	2 (8 %)	4 (16 %)	19 (76 %)	
Educational level of the mother (No. = 11)				
Junior high school	1 (50.00 %)	1 (50.00 %)	0	0.02
High school	0	1 (11.11 %)	8 (88.89 %)	
Educational level of the father (No. = 40)				
Junior high school	2 (8.00 %)	5 (20.00 %)	18 (72.00 %)	0.50
High school	0	4 (26.67 %)	11 (73.33 %)	
CHD	0	0	0	
BPD	1 (25.00 %)	1 (25.00 %)	2 (50.00)	0.10
Day-care attendance (No. = 44)				
Yes	0	3 (42.86 %)	4 (57.14 %)	0.25
No	2 (5.41 %)	6 (16.22 %)	29 (78.38 %)	
Exposure to ETS (No. = 43)				
Yes	1 (4.17 %)	4 (16.67 %)	19 (79.17 %)	0.72
No	1 (5.26 %)	5 (26.32 %)	13 (68.42 %)	
Breast feeding (No. = 15)				
Yes	0	2 (28.57 %)	5 (71.43 %)	0.51
No	1 (12.50 %)	1 (12.50 %)	6 (75 %)	

*CHD* congenital heart disease, *BPD* bronchopulmonary dysplasia, *ETS* environmental tobacco smoke

### Hospitalization and RSV^+^ in children evaluated with a chronological age of ≤12 or >12–24 months

**Fig. 2:**
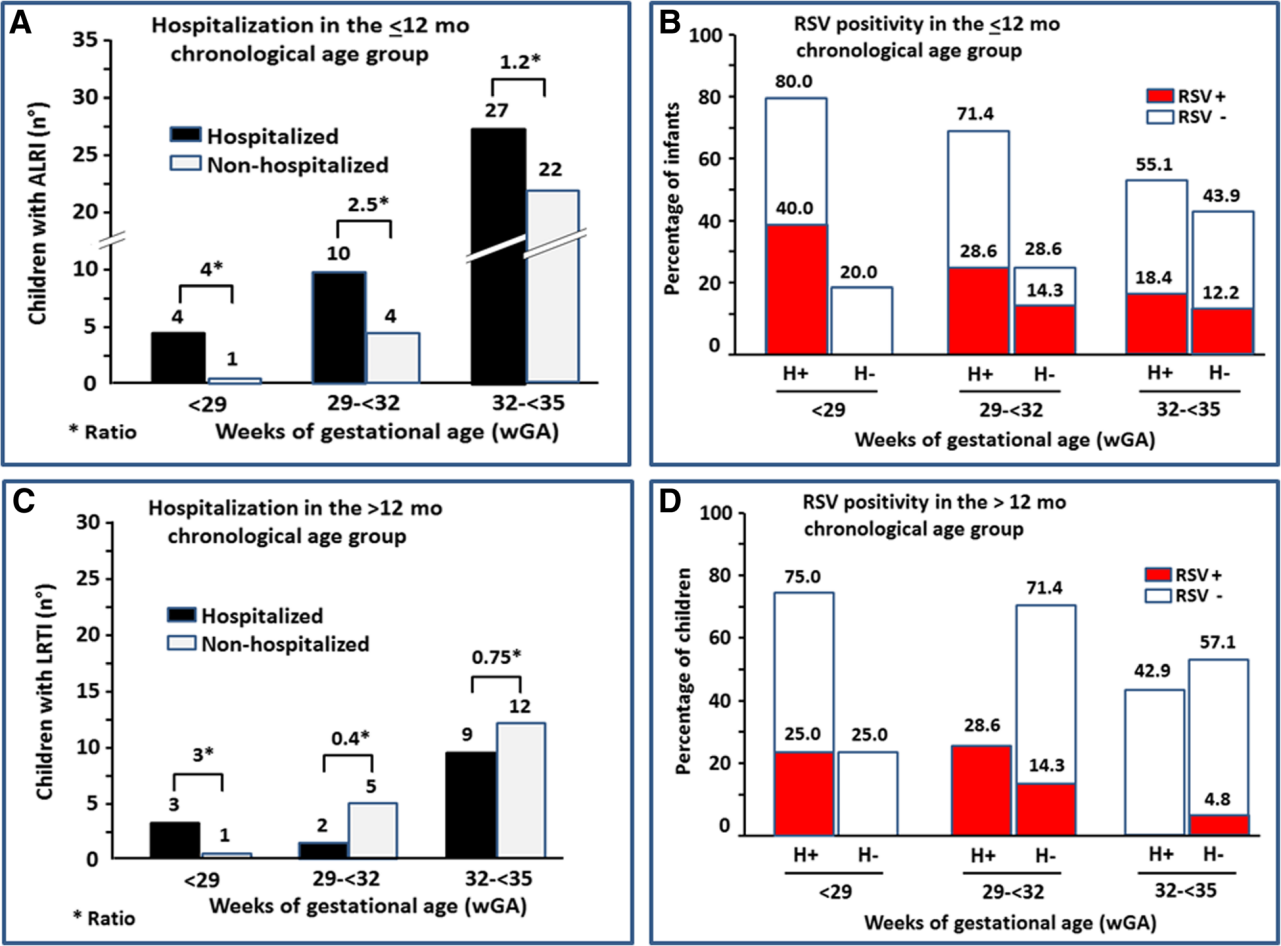
Data from the “Osservatorio” database: comparison between hospitalized (H^+^) and not hospitalized (H^−^) children. **a** On the ordinate, the number of H^+^ and H^−^ infants evaluated with a chronological age ≤12 months (mo) are shown and, on the abscissa, the three weeks’ gestational age (wGA) groups. The H^+^-to- H^−^ ratios in the three wGA groups are also included in the figure (*). **b** On the ordinate the percentage of H^+^ and H^−^ infants that were RSV+ or RSV- are shown and, on the abscissa, the three wGA groups. **c** and **d**. The same data in children evaluated with a chronological age >12–24 mo are shown. In the two chronological age groups, panels **a** and **c**, the tendency of the hospitalized-to-not-hospitalized ratio to progressively decrease with increasing wGA did not reach the statistically significance (*p* = 0.35 and *p* = 0.32, respectively). In the ≤12 mo group, panel **b**, the percentage of hospitalized infants that were also RSV^+^ was also progressively decreasing with increasing wGA, without reaching the statistically significance (*p* = 0.43). In the >12-24 mo, panel **d**, the low hospitalization numbers and hospitalized-to-not-hospitalized ratios were associated with a low RSV^+^ frequency in the <29 and 29- < 32 wGA groups, and no RSV^+^ hospitalized infants in the 32- < 35 wGA group (Fig. 2d) (*p* = 0.04)

Of the 100 premature infants referred to the emergency departments for ALRI, 68 were evaluated when they were ≤12 month of age: 7.4 % in the <29 wGA, 20.6 % in the 29- < 32 wGA and 72.1 % in the 32- < 35 wGA groups (Fig. 2a). Forty one (60.3 %) of these infants were hospitalized and the hospitalized-to-not-hospitalized ratio tended to progressively decrease with increasing wGA, being 4.0, 2.5 and 1.2, respectively in the three wGA groups (*p* = 0.35) (Fig. 2a). The percentage of hospitalized infants that were also RSV^+^ was also progressively decreasing with increasing wGA, being 40.0, 28.6 and 18.4, respectively in the three wGA groups (*p* = 0.43) (Fig. 2b). Of the 32 children evaluated who were >12 months of age, 12.5 % were in the <29 wGA, 21.9 % in the 29- < 32 wGA and 65.6 % in the 32- < 35 wGA groups (Fig. 2c). Fourteen (43.7 %) of these infants were hospitalized and the hospitalized-to-not-hospitalized ratio was positive in the <29 wGA group (3) but not in the 29- < 32 and 32- < 35 wGA groups (0.4 and 0.7) (*p* = 0.32) (Fig. 2c). The low hospitalization numbers and hospitalized-to-not-hospitalized ratios were associated with a low RSV^+^ frequency in the <29 and 29- < 32 wGA groups, and no RSV^+^ hospitalized infants in the 32- < 35 wGA group (*p* = 0.04) (Fig. 2d).

### Hospitalization and RSV^+^ in children evaluated with a chronological age of ≤6 or >6–12 months

**Fig. 3:**
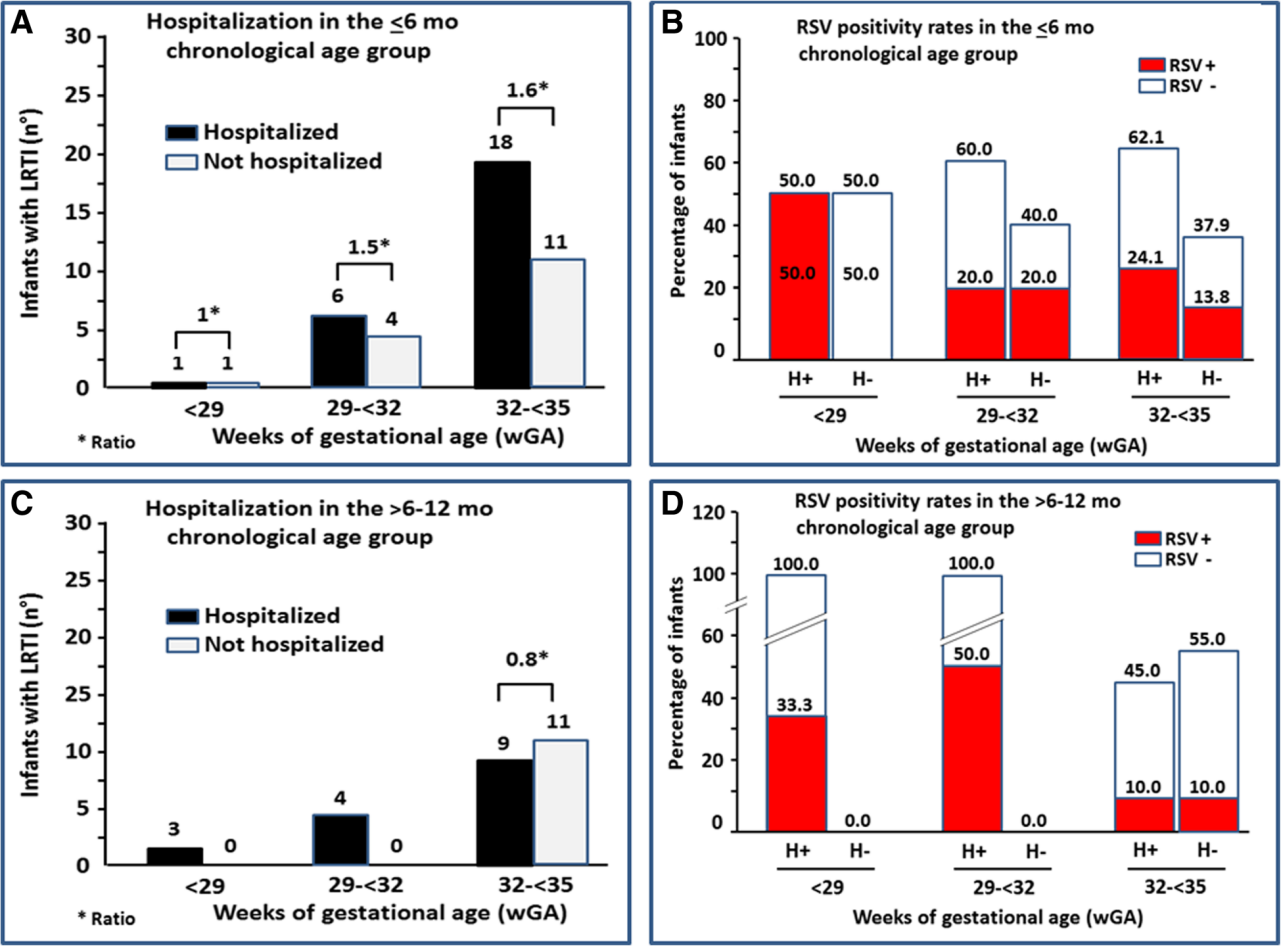
Data from the “Osservatorio” database: comparison between hospitalized (H^+^) and not hospitalized (H^−^) children. **a** On the ordinate, the number of H^+^ and H^−^ infants evaluated with a chronological age ≤6 months (mo) are shown and, on the abscissa, the three weeks’ gestational age (wGA) groups. The H^+^-to- H^−^ ratios in the three wGA groups are also included in the figure (*). Positive hospitalized-to-not-hospitalized ratios were detected in all wGA groups, with no statistically significant difference (*p* = 0.94). **b** On the ordinate the percentage of H^+^ and H^−^ infants that were RSV+ or RSV- are shown and, on the abscissa, the three wGA groups. **c** and **d**. The same data in children evaluated with a chronological age 6- > 12 mo are shown

The data related to the 68 infants evaluated with a ≤12 month age, were then divided in two subgroups, those with a ≤6 month age and those with a 6- ≤ 12 month age. Out of the 41 (60.3 %) infants with a ≤6 month age, 4.9 % belonged to the <29 wGA group, 24.4 % to the 29- < 32 wGA group and 70.7 % to the 32- < 35 wGA group (Fig. 3a). Positive hospitalized-to-not-hospitalized ratios were detected in all wGA groups, 1.0, 1.5 and 1.6, respectively, with no statistically significant difference (*p* = 0.94) (Fig. 3a) despite a low RSV^+^ frequency in the hospitalized infants, belonging to the 29- < 32 and 32- < 35 wGA groups (20.0 and 24.1, respectively) (Fig. 3b).

In the >6-12 month-age group, all infants with a <29 and 29- < 32 wGA were hospitalized (Fig. 3c); a relatively high RSV^+^ frequency was detected, mainly in the 29- < 32 wGA group (Fig. 3d). In contrast, in the 32- < 35 wGA group the hospitalized-to-not-hospitalized ratio was negative with a relatively low RSV^+^ frequency.

## Discussion

Because of the high social and financial healthcare burden and the difficulties in developing safe, clinically effective and financially affordable therapeutic measures, understanding the characteristics and the impact of neonatal RSV infection is important in planning preventive strategies. Prophylaxis with palivizumab is the only preventive measure available to reduce the incidence of RSV-induced hospitalizations in high-risk infants [17]. Although recent reports have consistently shown that RSV hospitalizations for premature children is highest in the first year of age [4–7, 17–24], very few studies have identified the chronological and gestational ages of RSV-specific hospitalizations more precisely in different nations [4, 18, 23, 24]. Because of different social and epidemiological factors, the eligibility criteria to receive palivizumab prophylaxis may be different in different countries, especially in late preterm infants.

In evaluating the Osservatorio database, we found that, consistent with premature infants in the general population, most of the children evaluated in the four RSV seasons were in the 32- < 35 wGA group [25]. This was true in the infants enrolled both in the first and in the second year of age. However, in the first year of age, the hospitalized-to-not hospitalized ratio was positive in all three wGA groups, progressively decreasing with increasing wGA. A similar trend was observed for RSV^+^ frequency, suggesting that, at least in the first year of age, the most premature infants were more vulnerable and prone to RSV infection. Very few <29 wGA infants with a ≤6 month age were included in the study, making sub-analyses in this group difficult. The instructions for the management, released after the hospital discharge, are particularly detailed for the very preterm infants and their parents are generally very careful in avoiding exposure to any factor that may facilitate infections [26]. The higher vulnerability of infants with ≤6 month age, belonging to the 29- < 32 and 32- < 35 wGA groups, is confirmed by the positive hospitalized-to-not hospitalized ratios (1.5 and 1.6, respectively), despite the relatively low RSV^+^ frequency. In the >6-12 month-age infants, positive hospitalized-to-not hospitalized ratios were detected in the <29 and 29- < 32 wGA groups, associated with a relatively high RSV^+^ frequency, whilst the 32- < 35 wGA group showed a negative hospitalized-to-not-hospitalized ratio and a relatively low RSV^+^ frequency. Interestingly, all the infants of the <29 and 29- < 32 wGA groups found to be RSV^+^ were hospitalized. Therefore not only a high vulnerability, but also an elevated predisposition to RSV infection seem to characterize the more premature infants in the second semester of life, when the risk of hospitalization seems to be particularly related to a more severe viral infection, i.e. RSV infection, not only in the <29 wGA, but also in the 29- < 32 wGA group.

The revised recommendations on palivizumab prophylaxis for RSV of the Italian Neonatology Society (Società Italiana di Neonatologia, SIN) recognize that is difficult to establish a gestational age “threshold” differentiating between high and low risk in premature neonates of less than 32 weeks GA [27]. It is undeniable that, as stated by the SIN recommendations and as also demonstrated by the present report, risks of RSV-related hospitalization decrease as gestational age increases and that the majority of hospitalizations because of RVS infection occurred during the first six months of life [27]. In relation to RSV prophylaxis, the SIN document recommends palivizumab for infants with <29 wGA and age ≤12 months at the beginning of the epidemic season, with a level of evidence II and a strength of recommendation A [27]. For infants with 29–35 wGA and age ≤12 months at the beginning of the epidemic season, with a level of evidence IV and a strength of recommendation B, the SIN document suggests to take in consideration palivizumab prophylaxis in presence of risk conditions predisposing to severe infections and/or need for hospitalization [27–31].

Evaluating preterm infants with ≤6 month of age in the Osservatorio database, we found positive hospitalization ratios both in the 29- < 32 and 32- < 35 wGA groups, whilst in the older infants (>6–12 month-age), only the 29- < 32 wGA group, but not the 32- < 35 wGA group, showed positive hospitalization ratios, with a 50 % RSV^+^ frequency. Evaluating the ≤12 and the >12 month-age groups it was clear that the trend in hospitalized-to-not hospitalized ratios showed a progressive decrease not only with increasing wGA but also increasing chronological age. Indeed in the older children, a positive ratio was detected only in the most premature group, the <29 wGA.

The major limitations of the present study are: a) the retrospective nature of the study but, we had no other options, since we wanted to evaluate data collected in years when palivizumab prophylaxis for RSV disease in premature infants was not widespread in our country; b) the low number of children born before <35 wGA included in this report, and the impossibility to include other young children in the evaluation of data related to a retrospective study, give reason for the lack of statistical significance, when the different groups and subgroups (wGA, age and risk factors) were compared; c) the number of children enrolled in the database could not be adjusted for the distribution of premature infants in the general population since we could not find the “live birth certificates” of all the Italian regions related to the 5 years of the study period.

## Conclusions

With the above limitations, the data on the hospitalized-to-not-hospitalized ratios and on RSV^+^ frequency in the first but also in the second semester of life, seem to show a remarkable vulnerability of the 29- < 32 wGA group infants. Although further studies are needed to identify risk factors that may increase the risk of severe RSV-induced ALRI in this population, the results of this retrospective analysis may be useful for prophylaxis planning, at least in our country.

### “Osservatorio VRS” study sites

Azienda Ospedaliero-Universitaria di Parma, Parma; Azienda Ospedaliera G. Salesi, Ancona; Istituto G. Gaslini, Genova; Ospedale San Paolo, Milano; Ospedale Santobono, Napoli; Ospedale dei Bambini Giovanni di Cristina, Palermo; Azienda Ospedaliera di Padova, Padova; Ospedale Pediatrico del Bambino Gesù, Roma; Ospedale Infantile Regina Margherita, Torino; IRCCS Burlo Garofolo, Trieste; Ospedale Infantile Anna Meyer, Firenze; Presidio Ospedaliero Annunziata, Cosenza; Presidio Ospedaliero Garibaldi, S. Luigi, S. Curro, Ascoli Tomaselli, Catania; Ospedale Maggiore, Bologna; Ospedale Civile di Legnano, Legnano.

## Abbreviations

ALRI: acute lower respiratory tract infection
RSV: respiratory syncytial virus
wGA: week gestational age
